# Iris cyst after femtosecond laser-assisted cataract surgery: a case report

**DOI:** 10.1186/s12886-021-01803-y

**Published:** 2021-01-13

**Authors:** Po-Ying Wu, Meng-Hsien Wu, Chi-Cheng Wu, Chi-Chin Sun

**Affiliations:** 1 Department of Medicine, College of Medicine, National Taiwan University, Taipei, Taiwan; 2 Cheng-Ching Eye Institute, Kaohsiung, Taiwan; 3 Department of Business Management, National Sun Yat-sen University, Kaohsiung, Taiwan; 4 Department of Medicine, College of Medicine, Chang Gung University, Taoyuan, Taiwan; 5 Department of Ophthalmology, Chang Gung Memorial Hospital, 222 Mai Chin Road, An Leh District, Keelung, Taiwan

**Keywords:** Iris cyst, Femtosecond laser-assisted cataract surgery (FLACS), Argon laser cystotomy, Case report

## Abstract

**Background:**

Secondary iris cysts are uncommon complication after cataract surgery. The reports of an iris cyst after conventional phacoemulsification surgery are scanty, let alone the iris cyst following femtosecond laser-assisted cataract surgery (FLACS). We herein report an unusual case of an iris cyst after an uneventful FLACS.

**Case presentation:**

A 64-year-old man who was healthy underwent FLACS for a moderate cataract of his left eye. Shortly after surgery, he achieved 20/20 vision, but anterior bowing of temporal iris was noted on postoperative day 9 with a retro-pupillary iris cyst at temporal-inferior quadrant found after pupil dilatation. The cyst was confirmed by ultrasound bio-microscopy afterward. Four weeks later, argon laser cystotomy was performed, and the cyst disappeared 3 days later. The patient’s vision remained stable thereafter.

**Conclusion:**

Although rare, secondary iris cyst may be one of the complications after FLACS. Argon laser cystotomy is effective in the management of post-FLACS iris cyst.

## Background

Iris cysts are uncommon and can be classified into two categories, primary and secondary, while secondary iris cysts are even less common than primary cysts [1]. Secondary cysts can be implantation cysts, originating by an invasion of conjunctival or corneal epithelial cells following surgical trauma or a penetrating wound [2]. These secondary iris cysts are thought to be the complications of large corneal incisions during surgeries, for instance, extra-capsular cataract extraction (ECCE), intra-capsular cataract extraction with vitreous loss (ICCE + VL), and penetrating keratoplasty [3].

Femtosecond laser-assisted cataract surgery (FLACS) has been widely used nowadays since it first introduced in 2008 [4]. The advantages of FLACS over conventional phacoemulsification surgery (CPE) are its precision and predictability. Although FLACS is thought to be a safe and trustable technique, it can still cause several post-operative complications, such as elevated intraocular pressure and macular edema [5]. Nevertheless, there is no report of secondary iris cyst after FLACS. Even in CPE, only 2 cases of iris cyst following CPE have been reported [6, 7]. The iris cysts in the two cases were found 2 months and 3 years postoperatively, respectively. Herein, we report an unusual case of an iris cyst presented shortly after an uneventful FLACS and was successfully treated by argon laser cystotomy.

## Case presentation

A 64-year-old man who was otherwise healthy underwent FLACS for a moderate cataract in his left eye due to the rapidly worsening of vision during outdoor and night driving. Preoperative ocular examination showed moderate nuclear sclerotic and posterior subcapsular cataract, relatively shallow anterior chamber depth (2.33 mm) with corrected distance visual acuity (CDVA) of 20/60 in the left eye and no other ocular pathology was noted.

Uneventful FLACS was performed using LenSx (Alcon, Fort Worth, TX, USA) with regular setting of circular continuous capsulorrhexis, fragmentation, and wound construction. Phacoemulsification with temporal approach using Centurion (Alcon, Fort Worth, TX, USA) was then carried out smoothly as routine surgery. Postoperative care and follow-up were unremarkable, and he gained uncorrected visual acuity of 18/20 on postoperative day one.

**Fig. 1:**
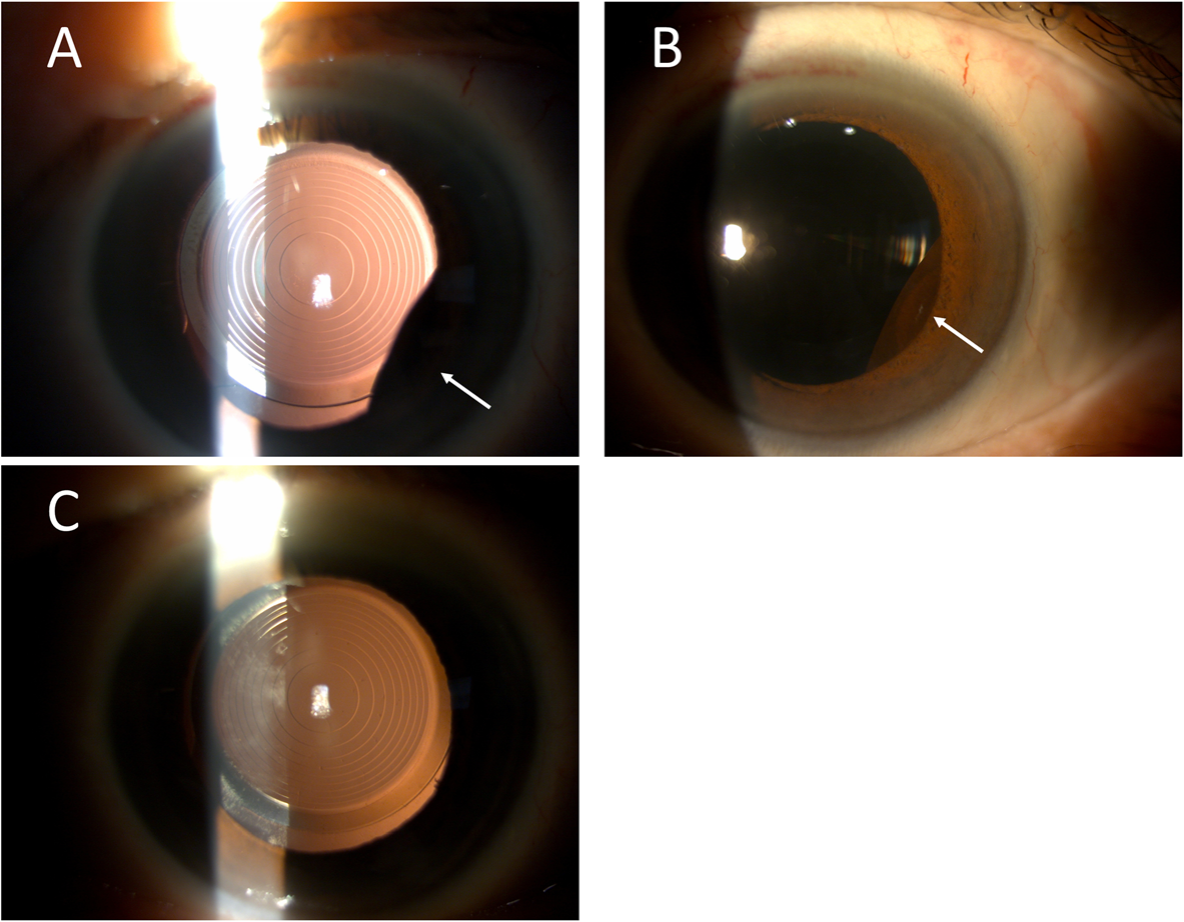
External eye photographs of the left eye in the 64-year-old patient. **a**, **b** A retro-pupillary iris mass at temporal-inferior quadrant (white arrows) was found after pupil dilatation nine days after FLACS. **c** The iris cyst disappeared three days after argon laser cystotomy, and the intraocular lens was still in good position

**Fig. 2:**
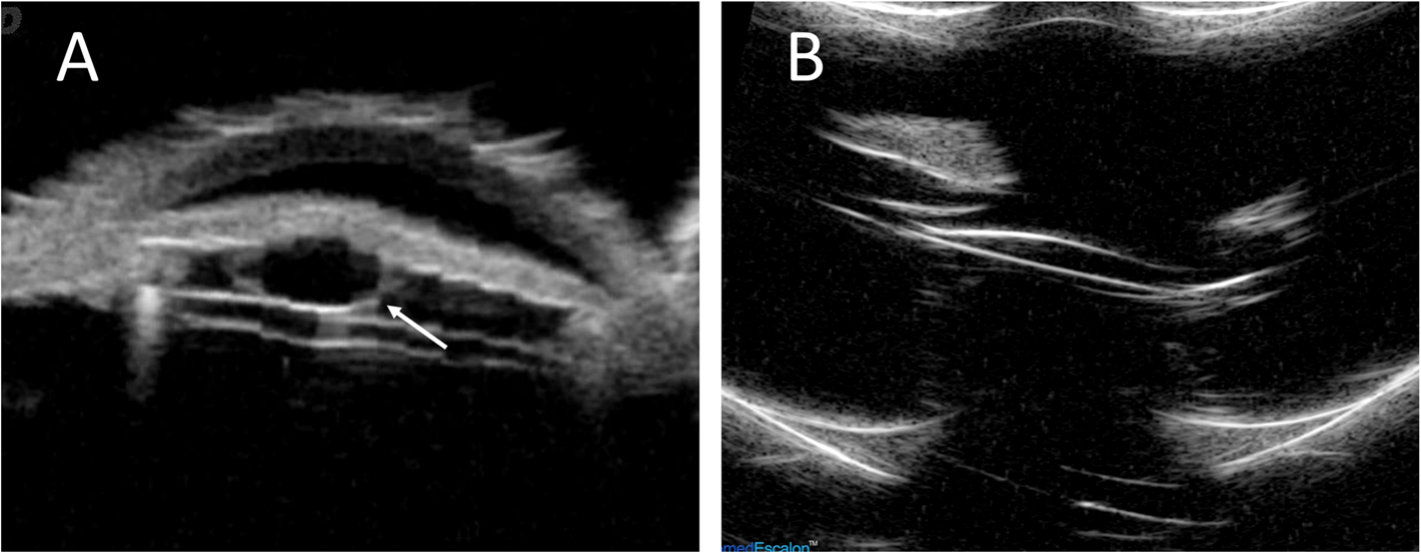
Ultrasound bio-microscopic images of the left eye in the 64-year-old patient. **a** Ultrasound bio-microscopy showed a typical hallow retro-pupillary cyst (white arrow). **b** Repeated ultrasound bio-microscopy confirmed no residual iris cysts 10 days after argon laser cystotomy

However, anterior bowing of iris and asymmetrically shallowing of the anterior chamber were noted on the temporal side without any visual disturbance 9 days after operation. An iris mass was found after pupil dilatation (Fig. 1a,b), but there was no other ocular abnormality and the intraocular lens remained in situ. A retro-pupillary iris cyst was identified by ultrasound bio-microscopy (Fig. 2a).

Despite the iris cyst, the postoperative CDVA of the left eye remained 20/20 during the follow-up period. Owing to not obvious cyst regression, argon laser cystotomy (50 um/0.2 sec / 500 mW/ 16 shots) was applied under the slit lamp one month later. The iris cyst became smaller 3 days after the procedure (Fig. 1c). Repeated ultrasound bio-microscopy demonstrated no residual iris cyst (Fig. 2b). The CDVA in his left eye was 20/20 and remained stable after cystotomy.

## Discussion

The pathogenesis of secondary cysts is that conjunctival or corneal epithelial cells invade into the anterior chamber following surgical trauma or a penetrating wound [2]. Iris, a vascularized tissue with rich nutrients, provides epithelial cells an ideal place for proliferation, resulting in an iris cyst. Histopathology of secondary implantation cysts is composed of multiple layers of non-keratinized stratified squamous epithelium [8]. Symptoms or signs of secondary iris cysts are varied. Iritis or uveitis is found to be the most common symptoms. A history of preceding surgery, trauma, or inflammation is nearly always present. For those iris cysts covering the visual axis, visual disturbance can also be the chief complaint of the patients.

It is possible that epithelial cells may be carried to anterior chamber by surgical instruments. This can explain why post-operative iris cysts have not been reported previously in FLACS owing to a corneal incision in FLACS created by femtosecond laser rather than a surgical instrument. Since formation of secondary iris cysts relates to epithelial downgrowth, the iris cysts should be uncommon in both FLACS and CPE due to small corneal incisions, theoretically. As a result, it is not surprising that the rare cases of iris cysts were reported after CPE [6, 7], let alone the report in FLACS postoperatively. One of the advantages of FLACS is that wound length created by femtosecond laser is also comparably small and precise as those created by keratomes. However, using femtosecond lasers can create more ideal clear corneal incisions with lower rates of endothelial gap, endothelial misalignment, and Descemet membrane detachment compared with those using metal keratomes [9]. This may also explain the low incidence of secondary iris cysts after FLACS.

However, adopting FLACS does not guarantee the freedom from iris cysts. We hypothesize that a subtle injury to iris during femtosecond laser application, subsequent phacoemulsification or any other steps might provide an adequate vascularized space for epithelial cells to colonize and proliferate. For example, iris injury may be induced during irrigation and aspiration. In either FLACS or CPE, this crucial step cannot be avoided. Previous study indicated that FLACS can reduce cumulative dissipated energy, which may result in the lower risk of anterior chamber inflammation [10]. We infer that less ultrasound energy may also reduce the damage to iris. Nevertheless, femtosecond laser pulse may directly harm the iris if the built-in positioning system does not properly work. Further studies are mandatory to investigate the influence of ultrasound or laser energy to iris or compare the damage to iris in FLACS and CPE.

It is also interesting that the onset of the iris cyst in our patient was only 9 days after FLACS. Other cases of a secondary iris cyst usually reported as late-onset from months to years after the surgery [2]. It could be explained by the easy accessibility of medical services in our country. Unlike other cases, our patient was found in the regular postoperative examination on day 9 without any discomfort or visual disturbance. We think that iris cysts in other cases may be appeared shortly but were not discovered until the occurrence of any symptoms.

Treatments for iris cyst include simple observation, fine-needle aspiration, laser (including argon or Nd:YAG laser), or surgical excision. Laser therapy has become the current trend because of minimal invasion. Secondary iris cysts possess a greater potential for associated complications compared with primary iris cysts [2]. For example, the coincidence of the phakic intraocular lens and the cyst may lead to potentially sight-threatening complications [11]. Argon laser cystotomy appears to provide an effective result for postoperative iris cysts [12]. Though we successfully applied the argon laser cystotomy to resolve the iris cyst after FLACS, however, long term follow-up is needed to observe possible complications of laser cystotomy including focal lens or corneal damage, retinal detachment, bleeding, elevated intraocular pressure, and progression of peripheral anterior synechia.

In conclusion, though FLACS is a safe and precise technique, pre-programmed corneal incisions in FLACS does not guarantee the freedom from iris cysts. Clinicians should carefully inspect the patients after FLACS since an iris cyst may be asymptomatic and shortly occur after surgery. Argon laser cystotomy is an effective tool for managing post-FLACS iris cyst.

## Data Availability

The datasets used and/or analyzed during the current study are available from the corresponding author on reasonable request.

## Abbreviations

FLACS: Femtosecond laser-assisted cataract surgery
ECCE: Extra-capsular cataract extraction
ICCE + VL: Intra-capsular cataract extraction with vitreous loss
CPE: Conventional phacoemulsification surgery
CDVA: Corrected distance visual acuity
